# Carbapenem-Resistant Enterobacteriaceae Posing a Dilemma in Effective Healthcare Delivery

**DOI:** 10.3390/antibiotics8040156

**Published:** 2019-09-20

**Authors:** Angus Nnamdi Oli, Chimaobi Johnpaul Itumo, Princeston Chukwuemeka Okam, Ifeanyichukwu U. Ezebialu, Kenneth Nchekwube Okeke, Christian Chukwuemeka Ifezulike, Ifeanyi Ezeobi, George Ogonna Emechebe, Ugochukwu Moses Okezie, Samson A. Adejumo, Jude Nnaemeka Okoyeh

**Affiliations:** 1 Department of Pharmaceutical Microbiology and Biotechnology, Faculty of Pharmaceutical Sciences, Agulu, Nnamdi Azikiwe University, Awka 420108, Anambra State, Nigeria; 2 Department of Pharmacology and Therapeutics, College of Health Sciences, Faculty of Medicine, Nnamdi Azikiwe University, Nnewi Campus, Nnewi 435101, Nigeria; 3 Department of Obstetrics and Gynaecology, Faculty of Clinical Medicine, Chukwuemeka Odumegwu Ojukwu University, Awka Campus, Awka 420108, Anambra state, Nigeria; 4 Department of Pediatrics, Faculty of Medicine, Nnamdi Azikiwe University, Nnewi Campus, Nnewi 435101, Anambra State, Nigeria; 5 Department of Pediatrics, Faculty of Clinical Medicine, Chukwuemeka Odumegwu Ojukwu University, Awka Campus, Awka 420108, Anambra State, Nigeria; 6 Department of Orthopaedic Surgery, Faculty of Clinical Medicine, Chukwuemeka Odumegwu Ojukwu University, Awka Campus, Awka 420108, Anambra State, Nigeria; 7 Department of Biology and Clinical Laboratory Science, Division of Arts and Sciences, Neumann University, One Neumann Drive, Aston, PA 19014-1298, USA

**Keywords:** carbapenem-resistance, *enterobacteriaceae*, carbapenemase, carbapenems, Super-bugs, Gram negative organisms

## Abstract

The emergence and spread of Carbapenem-resistant *Enterobacteriaceae* (CRE) is seriously posing threats in effective healthcare delivery. The aim of this study was to ascertain the emergence of CRE at Chukwuemeka Odumegwu Ojukwu University Teaching Hospital (COOUTH) Awka. Biological samples were collected from 153 consenting patient from 5 clinics in the hospital. The isolates were identified using standard microbiological protocols. Susceptibility to meropenem was done using Kirby-Bauer disc diffusion method on Mueller Hinton Agar. A total of 153 patients were recruited in this study. About one half of those from rural, 63.64% from Sub-urban and 42.27% from urban areas had significant *E. coli* and *Klebsiella* spp infections. The male: female ratio of the *Enterobacteriaceae* infection was 1:1. Almost as much inpatient as outpatient study participants had the infections. The infections were observed mostly on participants with lower educational status. The unmarried individuals were most infected compared to their married counterparts. *Enterobacteriaceae* infection rate was 50.98%. Of this, 28.21% had CRE infection while the overall prevalence of the CRE in the studied population was 14.38% (22/153). This study shows that CRE is quickly emerging in both community and hospital environments. *Klebsiella* spp was the most common CRE in this hospital especially *Klebsiella oxytoca*. Hospitalization was a strong risk factor in the CRE infections. Rapid and accurate detection is critical for their effective management and control.

## 1. Introduction

*Enterobacteriaceae* are a large family of aerobic Gram-negative rods existing mostly as part of normal flora in the human and animal colons. More than other Gram negative organisms, they are particularly of clinical important in the cause of nosocomial and community acquired bacterial infections [1]. The mainstay for the treatment of *enterobacteriaceae* infections is the beta-lactam group of antibiotics [2]. Carbapenems are beta-lactams antibiotics used for the treatment of infections known or suspected to be caused by multidrug-resistant (MDR) bacteria. Like other beta-lactams antibiotics, they act by binding to penicillin-binding proteins, thereby inhibiting the synthesis of bacterial cell wall. They are primarily used in hospitalized patients. Multidrug resistant, extensively drug-resistant (XDR) and pandrug-resistant (PDR) organisms are rapidly emerging and spreading worldwide [3,4] and at a rate extremely faster than the development of new drug molecules. They have been implicated in community-acquired as well as hospital-acquired infection outbursts such that among the most important contemporary healthcare issues are the emergence and spread of antimicrobial-resistant infections [4,5]. Also, that Carbapenem-resistant *Enterobacteriaceae* (CRE) infections are with us now has been a global health challenge and constitutes much concern to public and private health services [6]. Early and efficient detection of CRE in human and animals hosts are critical in controlling the infection and their spread [7]. Carbapenem resistance in *enterobacteriaceae* may result from one or more of such mechanisms including but not limited to [7,8,9]:

- production of carbapenemase enzymes (Carbapenem-hydrolysing enzymes) namely: the class A carbapenemases (the *Klebsiella pneumoniae* carbapenemase types), the class B or Metallo-beta-lactamases (MBLs), and class D Oxacillinases (e.g., OXA-48-like enzymes)
- production of extended spectrum beta-lactamases (ESBLs)
- production of AmpC enzymes (mostly, plasmid-mediated)
- porins loss leading to drug decreased permeability

For many clinicians in Nigeria, carbapenems are the preferred option for treating infections due to multidrug‑resistant *enterobacteriaceae*. The emergence and spread of carbapenemase-producing *enterobacteriaceae* (CPE) is seriously posing threats in effective healthcare delivery. There is need for a continuous epidemiological monitoring of the prevalence of these Carbapenem-resistant *enterobacteriaceae* isolates. This will aid evidence-based decision for limiting the spread and controlling the infections. It will also serve as a useful baseline for determining the rational and effective chemotherapeutic option for the management of Carbapenem-resistant *enterobacteriaceae* in the center and will serve as a formidable approach to the control of antimicrobial resistance. The aim of this study was to ascertain the emergence of CRE in the tertiary health institution. No results, as yet, exist and clinicians have no scientifically reported proof of the existence of Carbapenem-resistant *enterobacteriaceae* infections in the facility. This study will enable them understand a rational approach to tackle such infections and help offer ways to curtail them. The knowledge of the incidence of organisms harboring the carbapenemase enzymes will aid in rational prescribing and patients’ education.

## 2. Results

**Figure 1:**
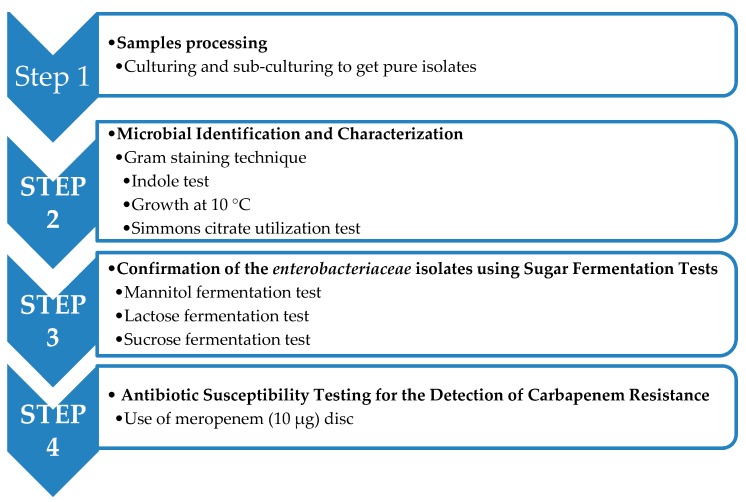
A flowchart of the detection of the Carbapenem-resistant *enterobacteriaceae*.

The samples were first processed and the isolates identified (Figure 1). The identified *enterobacteriaceae* were confirmed by biochemical methods while the confirmed isolates were tested for carbapenemase production using Meropenem (10 µg) disc. Resistance to Meropenem (10 µg) was taken as Carbapenem-resistant *enterobacteriaceae*. 

### Characteristics of Study Population

A total of 153 patients were recruited in this study (Table 1) and 49.06% of those from rural, 63.64% from Sub-urban and 42.27% from urban areas had significant *E. coli* and *Klebsiella* spp infections. The male: female ratio of the *enterobacteriaceae* infections was 1:1. Almost as much inpatient as outpatient study participants had the infections. The infections were observed mostly on participants with lower educational status. The unmarried individuals were most infected compared to their married counterparts.

The prevalence of *enterobacteriaceae* in the samples studied was 50.98% (78/153) out of which 28.21% (22/78) showed resistance to Carbapenem (meropenem) antibiotic. Although the percentage of CRE isolated was high (14.38% i.e., 22/153), it is not statistically significant (*p* value > 0.05). Majority of the CRE were *K. oxytoca* (9/22). Out of the 12 *K. oxytoca* isolates, 75% were CRE. A 2-Way ANOVA analysis of the result showed that the chance of isolating *enterobacteriaceae* is similar at all the locations of residence of the study participants. The species of *enterobacteriaceae* isolated accounted for 72.44% of the total variance in infection rate. The *p* value = 0.0342. The species of *enterobacteriaceae* isolated is considered very significant in relation to infection rate. Also, the location of residence of the study participants accounted for 11.11% of the total variance in infection rate. The *x* value = 0.3561. Location has no effect overall, and so the effect is considered not quite significant. Gender has no effect on infection rate of *enterobacteriaceae*. Chi-square value = 0.9354, and *p* value = 0.6265. It is therefore concluded that both sexes have equal risks of being infected. Also, Chi-square test for trend of CRE among the study participants showed that it is not significant statistically (*p* value = 0.7811).

Two-Way analysis of the prevalence of the infection by age of the patients showed that the *Enterobacteriaceae* isolates had the same effect at all levels of age groups of patients. *Enterobacteriaceae* isolates accounted for 22.21% of the total variance among the age groups. The *p* value = 0.0506 and so the effect is considered not quite significant. However, age of patients accounted for 64.76% of the total variance in infection rate with a *p* value = 0.0096. The effects of age on infection rates are considered very significant. However, patients’ age did not affect CRE infections rate.

Two-Way analysis of the prevalence of the infection by the patients’ marital status showed that the *Enterobacteriaceae* isolates had the same effect at all levels of the patients’ marital status. *Enterobacteriaceae* infections accounted for 13.04% of the total variance seen in the patients’ marital status with a *p* value = 0.1227. The effect is considered not significant. However, marital status accounted for 74.08% of the total variance seen in the infection with a P value = 0.0067. The patients’ marital status significantly affected the prevalence of the *enterobacteriaceae* infections.

Patients’ literacy level accounted for 63.58% of the total variance in *enterobacteriaceae* infection rate with a *p* value = 0.0186. Literacy level, therefore, has some effect overall, and is considered significant.

Considering the infection rates of both *enterobacteriaceae* and CRE in relation to in-patient and out-patient settings, the Chi-square and Fisher’s exact tests respectively showed a *p* value < 0.0001 each. It is therefore concluded that there is a significantly higher relative risk of *enterobacteriaceae* and CRE infections in hospital setting (in-patients) compared to out-patients. 

Table 2 shows infection prevalence by the kind of samples collected. The results of the prevalence of the *enterobacteriaceae* isolated in relation to the kind of samples studied showed that the chance of isolating *enterobacteriaceae* is similar across all the samples. The species of *enterobacteriaceae* isolated accounted for only 11.97% of the total variance in infection rate with a P value of 0.7982. The species of *enterobacteriaceae* isolated is considered not significant in relation to infection rate with respect to sample type. 

The surgical wards and chest clinic contributed most to the resistant isolates (Table 3) while the out-patient clinic, gynecology and antenatal clinics as well as urine samples contributed list. However, there is no significant difference (*p* > 0.05). 

**Figure 2:**
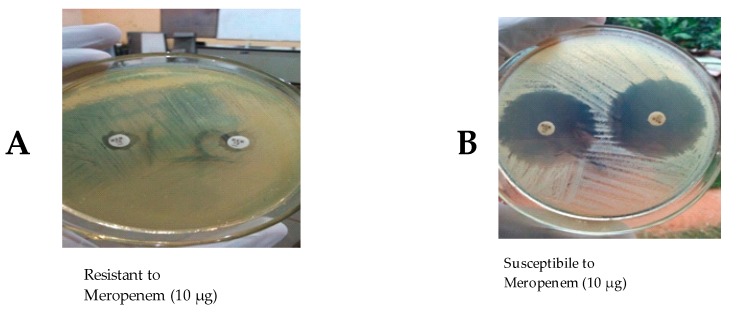
Antibiotic Susceptibility testing for the Detection of Carbapenem resistance.

Meropenem—a representative Carbapenem—is shown phenotypically to be liable to carbapenemase enzyme (Figure 2A) produced by isolates and so the isolates were resistant to the Carbapenem. In Figure 2B, the isolate was non-carbapenemase producer and so was susceptible to meropenem. 

A great percentage (28.21%) of the *enterobacteriaceae* isolates was resistant to Carbapenem (Table 4). There was a significant difference in the susceptibility profile of the isolates, Chi-square = 15.36 and *p* value = 0.0005. 

## 3. Discussion

In this study, a total of 78 *Escherichia coli* and *Klebsiella* spp were isolated from clinical samples while the prevalence of Carbapenem resistance was 14.38% (22/153) in the hospital. This is quite high for a drug used as last resort in infection treatment. *Klebsiella oxytoca* was been reported as emerging *enterobacteriaceae* in hospital community and had shown considerable resistance to antibiotics [10,11,12]. The prevalence Carbapenem-resistant *enterobacteriaceae* found in our study is higher than that recorded in Kano, northern Nigeria (11.8%) [13]; Pune, India (1.6%) [14] and Morocco (2.5%) [15]; similar to that observed in Lagos, western Nigeria (15.2%) [9]; but lower than the report from Uganda (18.4%) [16]. The most common species of the CRE were *K. oxytoca* (41%, 9/22), followed by *K. pneumoniae* (32%, 7/22) and then *E. coli* (27%, 6/22). Contrary trend was observed in a study carried out in Turkey where *K. pneumoniae* was the most common specie [17]. 

While our study showed that of the factors investigated, only literacy levels (*p* value = 0.0058) and hospitalization (*p* value < 0.0001) actually affected the rate of CRE infections; the infection rates of *enterobacteriaceae* generally were found to be affected by factors such as age, marital status, level of education and hospital setting. Other studies proved that younger age group and hospitalization are common risk factors for Gram-negative *enterobacteriaceae* (GNE) infections [18,19] while hospitalization remains the leading cause of CRE infections [20,21,22]. Since hospitalization is a strong risk factor in CRE colonization; clinicians, other health workers and in fact, everyone should join hands to reduce patients’ length of stay in hospitals as this will reduce the risk of spread of CRE as well as control them. 

Sputum sample has the highest prevalence of CRE 15% which was in line with that of a study carried out in Indonesia [23] but at variance with that carried out in northern Nigeria where urine had the highest number of CRE [13]. All the resistant isolates in the sputum samples were *K. pneumoniae* which are well known for causing pneumonia and nosocomial respiratory tract infections [24]. The chest clinic (where these sputum samples were collected) had the highest prevalence rate of CRE (15%) followed by the surgical ward (11.54%). Surgical wards and intensive care units had been established by studies as one of the clinics in hospital where CRE are found, may be because of long term catheterization of bed ridden patients which is one of the risk factors implicated in the infection [21,25]. These infected patients serve as reservoirs for spreading the infection and contaminating the environment. The prevalence of CRE among inpatients 11.11% (17/153) was more when compared to that among outpatients 3.27% (5/153) in the hospital. This also shows that CRE can be both hospital and community acquired and that the transmission of CRE infection is more likely in hospitals [20,21,22]. 

A greater number of *enterobacteriaceae* isolates observed in the study came from urine sample (39.74%) followed by sputum (25.64%), wound/pus (19.23%) and anal swab (15.38%). In a comparable study, it was found out that the distribution of the sources of isolates had urine sample as the highest followed by blood while wound discharge and sputum were the least [26]. Urine sample being the highest source of isolates in each case could be due to urinary tract infection which accounts for almost 40% of nosocomial infection [27]. The total number of isolates that showed intermediate susceptibility to carbapenems (20.5%) is of clinical importance as some of them may have the inherent ability of producing carbapenemase enzymes and this may be an indicative of low level resistance which if left undetected may escalate to high level resistance [10,28]. The mechanisms of antimicrobial resistance [29,30] and virulence [31] among Gram-negative *enterobacteriaceae* and among CRE vary widely (7,8,9). Also, it is not impossible that there could be cross-resistance between the carbapenems, the penicillins and the cephalosporins as they are all beta-lactam antibiotics and are dipeptide mimics [32]. The carbapenems resistance has been attributed to intrinsic and/or acquired resistance mechanisms [7,33].

## 4. Materials and Methods

### 4.1. Study Area and Site, Study Population, and Ethical Clearance

The study was carried out from May to July 2017 at Chukwuemeka Odumegwu Ojukwu teaching hospital (COOUTH) Amaku, Anambra state in south east Nigeria. The hospital serves a mixed population—including the urban community of the State capital and large number of rural communities; the low-income, middle-income and high-income populations. It is also a tertiary health institution and is very strategic to the healthcare needs of the state. It provides medical care to patient as well as served for the undergraduate and post-graduate medical training and the training of pharmacy and nursing students. 

The study population included 57 inpatients and 96 outpatients (Total 153) who gave their informed consent, aged (1–80) years and are not on antibiotic for at least two weeks before sample collection. Sampling points included: antenatal and gyneacology clinic, Chest clinic, male and female surgical wards and peadiatric medical and surgical wards. Study protocol was approved by the ethical committee of the hospital (Approval Code: COOUTH/AA/VOL.X1/045 of 28th March 2017). 

### 4.2. Sample Size Calculation

The total sample size was obtained with the assumptions of power 80%, a confidence interval of 95% and a 5% level of significance (alpha = 0.05). A 10% attrition rate was added in order to get the total sample size of 153 study participants. Report created by GraphPad StatMate version 2.00 on 23-Jan-17 3:28:23 PM.

### 4.3. Microbiological Investigations

#### Sample Processing

The working bench was first disinfected with 70% alcohol and the Bunsen burner lit in order to create aseptic environment. The samples were separately and aseptically inoculated on to previously prepared and labeled sterile nutrient broth in test tubes. The tubes were properly with sterile cotton wool and then incubated at 37 °C for 24 h. The inocula that had growth were aseptically inoculated onto already prepared and sterilized MacConkey agar plates and then incubated at 37 °C for 24 h. Separate colonies of the isolates were sub-cultured in sterile nutrient agar plates and incubated aerobically at 37 °C for 24 h. This enabled the isolation of pure cultures.

### 4.4. Microbial Identification and Characterization

#### 4.4.1. Gram Staining Technique

Smears of the isolates were made on clean grease-free slides, air-dried, and then heat-fixed by passing across a burning flame. These were covered with Giemsa stain for 30–60 s and rinsed off with clean water. Then, Lugol’s iodine was added for 30–60 s and rinsed off with clean water. Next, 70% alcohol was used to decolorize the stain, and the sample was allowed to dry. Safranin red was used to counter-stain for 2 min, and the sample was rinsed off with clean water. The backs of the slides were cleaned with dry filter paper and, thereafter, the fronts were examined using an oil immersion microscope.

#### 4.4.2. Biochemical Tests

Series of biochemical tests were conducted to confirm the results obtained from the macroscopic and microscopic examinations of the isolates. However, only the tests with positive results are reported. 

#### 4.4.3. Indole Test

Peptone water (Oxoid, UK) was prepared according to the manufacturer’s specification and sterilized in an autoclave at 115 °C for 15 min. The test organisms were inoculated into test tubes containing 3 mL of the sterile peptone water and were incubated at 37 °C for 48 h. After incubation, 0.5mL of Kovac’s reagent (Sigma-Aldrich) was added to the test tubes. The production of a red color on the surface layer of the test tube within 10 min suggested the presence of *Escherichia coli*, *Proteus* spp., and *Klebsiella* spp.

#### 4.4.4. Confirmation of the Enterobacteriaceae Isolates Using Sugar Fermentation Tests

This was done using Mannitol Fermentation Test as described previously [34]. *Enterobacteriaceae* ferment mannitol to change the colour of the broth from red to yellow, indicating acid-gas production.

Again, lactose fermentation was performed as described previously [34] to differentiate lactose fermenting *Enterobacteriaceae* (such as *Escherichia coli* and *Klebsiella* spp) from non-lactose fermenters (such as *Salmonella* spp and *Shigellae* spp). All the isolates gave positive results.

The isolates were subjected to sucrose fermentation. Colour changes show presence of *Klebsiella pneumoniae* and absence of *E. coli.*

#### 4.4.5. Growth at 10 °C

The Indole-positive, mannitol-fermenting isolates were subjected to growth at 10 °C. The isolates that grew at this temperature after 48 h of incubation were confirmed as *Klebsiella oxytoca*.

#### 4.4.6. Simmons Citrate Utilization Test 

This test differentiates Enterobacteriaceae based on the utilization of citrate and ammonium dihydrogen phosphate as the only carbon and nitrogen sources, respectively. Slants of Simmons citrate agar (HiMedia Laboratories, India) were prepared in a bijou bottle following the manufacturer’s specification. A sterile straight-wire loop was used to firstly streak the test organism on the slant, and then it was stabbed. The slants were incubated at 35 °C for 48 h and were observed for a bright blue color. The *Klebsiella* spp. were citrate-positive, but *E. coli* was citrate-negative. Citrate-positive isolates were further incubated for 10 days and observed for H_2_S production. A positive result confirmed *K oxytoca*. 

#### 4.4.7. Storage of the Isolates

The colonies of the isolated and characterized isolates were stored in well-labeled double-strength nutrient agar slants in bijou bottles and kept in a refrigerator. 

#### 4.4.8. Antibiotic Susceptibility Testing for the Detection of Carbapenem Resistance

The bacteria isolates were subjected to Meropenem (10 µg) using the Modified Kirby-Bauer Susceptibility testing technique. The decision to use Meropenem to determine Carbapenem resistance was based on the fact that it offers the best balance between sensitivity and specificity when detecting carbapenemase producers in clinical setting [10,35,36]. The pure isolates were first inoculated into sterile nutrient broth and incubated at 37 °C for 24 h. The inocula were standardized by sub-culturing a loop full into 3 mL sterile nutrient broth and incubating for 1–3 h to allow for growth of organism till the turbidity was equivalent to the turbidity of 0.5 McFarland. The standardized inocula were swabbed aseptically on to previously prepared and sterilized Mueller Hinton Agar plates and were allowed to stand for about 5 minutes. Thereafter, Meropenem (10 µg) disks were aseptically pressed gently down on to the surface of the media, ensuring proper lap with the media. After 24 h incubation at 37 °C, the isolates with inhibition zone diameters of <16 mm were classified as Carbapenem resistant [37].

### 4.5. Statistical Data Analysis and Interpretation

The data were analysed using GraphPad Prism version 5.00 for Windows, GraphPad Software, Inc. San Diego California USA, www.graphpad.com. One-Way Analysis of Variance (ANOVA) was used to establish the mean differences in prevalence of the isolates among various age groups while the Chi Square checked for the relative risks of *enterobacteriaceae* infections in relation to gender and setting (hospital versus community). Two-Way Analysis of Variance (ANOVA) was used to establish the mean differences in infection rates of the different strains of the enterobacteriaceae isolated. Bonferroni post-tests were used to compare the effects of patients’ place of residence, Age, Marital status, educational level on infection rates. All P values reported are for a two-tailed test. The significance level was chosen at *p* < 0.05.

## 5. Conclusions

This study shows that Carbapenem-resistant *enterobacteriaceae* are quickly emerging in both community and hospital environments. *Klebsiella* spp. are the most common species resistant to Carbapenem in this hospital, especially *Klebsiella oxytoca*. Hospitalization is a strong risk factor in CRE infections. A closer eye should be kept in order to quickly diagnose and to control the CRE infections. Rapid and accurate detection, as well as a reduction in the length of hospital stay, are critical to their effective control. 

## 6. Patients

Patient recruitment and specific sample collection were done by the medical doctors in each clinic/study site. For the sputum and urine samples, appropriate sterile containers were issued to the patients, who were educated on how to collect the samples (i.e., productive cough and clean catch mid-stream urine, respectively). The specimens used for the study included wound swab/pus, anal swab, urine, and sputum containing cough. The demographic data of the patients were collected using a validated questionnaire.

## Figures and Tables

**Table 1:** Prevalence of the *enterobacteriaceae* isolated (Total number of Samples *N* = 153).

Variable	Patients Tested	Number with Infection (% Infected)			Number of Resistant Organisms * (% Resistant)		
**Location (residence)**		***K. p.*** **37 (24)**	***K. o.*** **12 (9)**	***E. coli*** **29 (19)**	***K. p.*** **7 (19)**	***K. o.*** **9 (75)**	***E. coli*** **6 (21)**
Rural	67	17 (25)	3 (4)	11 (16)	2 (12)	3 (100)	3 (27)
Sub-Urban	33	9 (27)	4 (12)	8 (24)	3 (33)	3 (75)	1 (25)
Urban	53	11 (21)	5 (9)	10 (19)	2 (18)	3 (60)	2 (20)
**Sex**							
Male	75	21 (28)	6 (8)	13 (17)	3 (11)	6 (100)	2 (15)
Female	78	16 (21)	6 (8)	16 (21)	4 (25)	3 (50)	4 (25)
**Age Group (yrs)**							
1–20	21	6 (29)	1 (5)	3 (14)	2 (33)	1 (100)	1 (33)
21–40	81	20 (25)	6 (7)	16 (20)	3 (15)	4 (67)	1 (6)
41–60	32	7 (22)	3 (9)	7 (22)	1 (14)	3 (100)	2 (29)
61–80	18	4 (22)	2 (11)	3 (17)	1 (25)	1 (50)	2 (67)
**Marital status**							
Single	63	16 (25)	4 (6)	15 (24)	2 (13)	4 (100)	3 (20)
Widowed	6	1 (17)	0 (0)	1 (17)	1 (100)	0 (0)	1 (100)
Divorced	0	0	0	0	0 (0)	0 (0)	0 (0)
Still Married	84	20 (24)	8 (10)	13 (15)	4 (20)	5 (63)	2 (15)
**Highest Education Completed**							
No formal Education	9	3 (33)	0 (0)	1 (11)	2 (67)	0 (0)	1 (100)
Primary school	24	4 (17)	2 (8)	5 (21)	1 (25)	2 (100)	1 (20)
Senior Secondary	66	20 (33)	3 (5)	15 (23)	1 (5)	2 (67)	2 (13)
Tertiary Education	47	10 (21)	6 (13)	7 (15)	3 (30)	4 (67)	1 (14)
Higher Degree	7	0 (0)	1 (14)	1 (14)	0 (0)	1 (100)	1 (100)
**Clinic Setting**							
In patients	57	6 (11)	10 (18)	16 (28)	5 (83)	7 (70)	5 (31)
Out patients	96	31 (32)	2 (2)	13 (14)	2 (6)	2 (100)	1 (8)

* Note: Both intermediate susceptibility and resistant strains are classified here as Carbapenem-resistant *enterobacteriaceae*.

**Table 2:** Percentage (%) Prevalence of Isolates in the samples.

Enterobacteriaceae Isolated	Number of Samples				Total = 153
	Urine *N* = 55	Sputum *N* = 51	Anal Swab *N* = 13	Wound/Pus Swab *N* = 34	
***E. coli***	12 (21.82)	2 (3.92)	10 (76.92)	5 (14.71)	29 (18.95)
***Klebsiella pneumoniae***	15 (27.27)	18 (35.29)	1 (7.69)	3 (8.82)	37 (24.18)
***Klebsiella oxytoca***	4 (7.27)	0 (0.00)	1 (7.69)	7 (20.59)	12 (7.84)
**Total**	31 (39.74)	20 (25.64)	12 (15.38)	15 (19.23)	78 (50.98)

**Table 3:** Carbapenem susceptibility of isolates.

A. **CLINIC SOURCES OF THE SAMPLES**				
**Clinics**	**Resistant (%)**	**Intermediate (%)**	**Susceptible (%)**	**Total (%)**
GOPD	0 (0.00)	6 (33.33)	12 (66.67)	18 (23.08)
Gyn. & Antenatal clinic	0 (0.00)	1 (12.50)	7 (87.50)	8 (10.26)
Medical Ward	0 (0.00)	2 (33.33)	4 (66.67)	6 (7.69)
Surgical Ward	3 (11.54)	5 (19.23)	18 (69.23)	26 (33.33
Chest Clinic	3 (15.00)	2 (10.00)	15 (75.00)	20 (25.64)
Total	6 (7.69)	16 (20.51)	56 (71.79)	78 (100)
B. **ANATOMICAL SITE SOURCES OF THE SAMPLES**				
Urine	0 (0.00)	9 (29.03)	22 (70.97)	31 (39.74)
Wound/Pus swab	2 (13.33)	4 (26.67)	9 (60.00)	15 (19.23)
Sputum	3 (15.00)	2 (10.00)	15 (75.00)	20 (25.64)
Anal swab	1 (8.33)	1 (8.33)	10 (83.33)	12 (15.38)
Total	6 (7.69)	16 (20.51)	56 (71.79)	78 (100)

**Table 4:** Carbapenem susceptibility of the Enterobacteriaceae Isolated.

Organism	Resistant	Susceptible	Total
***E. coli***	6 (20.69)	23 (79.31)	29 (37.18)
***K. pneumoniae***	7 (18.92)	30 (81.08)	37 (47.44)
***K. oxytoca***	9 (75.00)	3 (25.00)	12 (15.38)
**Total**	22 (28.21)	56 (71.79)	78 (100)
